# Assembly and comparative analysis of the complete mitochondrial genome of the spice plant *Cinnamomum longepaniculatum*

**DOI:** 10.1186/s12870-025-06839-6

**Published:** 2025-07-16

**Authors:** Yanling Fan, Linxin Tan, Ruizhang Feng, Xin Zhao, Xiangdong Xu

**Affiliations:** 1 The Faculty of Agriculture, Forestry and Food Engineering, Yibin University, Yibin, China; 2 Sichuan Province Engineering Technology Research Center of Oil Cinnamon, Yibin University, Yibin, China

**Keywords:** Mitochondrial genome, *Cinnamomum longepaniculatum*, Repeat sequence, Phylogenetic analysis, Linear structure

## Abstract

**Background:**

*Cinnamomum longepaniculatum*(Gamble) N. Chao ex H. W. Li (*C. longepaniculatum*), a species renowned for its leaves and twigs yielding essential oils, is extensively utilized as a vital raw material in traditional Chinese medicine, spice, and daily chemical products, and thus possesses both high economic value and significant scientific research value. However, to date, no detailed information on the mitochondrial genome (mitogenome) has been reported.

**Results:**

The mitogenome of *C. longepaniculatum* was characterized by a linear structure, spanning 870,686 bp with 46.94% GC content. It encompassed 44 protein-coding genes (PCGs), 28 tRNAs, and 3 rRNAs. Numerous repetitive sequences were observed, including 317 simple sequence repeats (SSRs), 52 tandem repeats and 874 pairs of dispersed repeats. Phylogenetic analysis based on the 41 conserved mitochondrial PCGs of *C. longepaniculatum* reflected its close evolutionary affinity to *C. camphora*, followed by *C. chekiangense* and *C. insularimontanum*. Furthermore, dot-plot analysis revealed diverse homologous collinear regions between *C. longepaniculatum* and other species belonging to the Laurales. Specifically, the largest collinear blocks, spanning over 861 kb, accounted for 95.59% of the total proportion between *C. longepaniculatum* and *Cinnamomum camphora*, indicating a high degree of conservation and similarity in their mitogenome structures.

**Conclusions:**

This study presents the initial assembly and annotation of *C. longepaniculatum* mitogenome, thereby enriching the limited repository of mitogenome sequences available for Laurales plants. These findings provide crucial molecular evidence for further studies on the evolutionary relationships and genomic evolution within the Laurales.

**Supplementary Information:**

The online version contains supplementary material available at 10.1186/s12870-025-06839-6.

## Background

The family Lauraceae, a significant group within the order Laurales of the Magnoliidae, encompasses approximately 60 genera and 3500 species, accounting for one-third of the Magnoliid plants [1]. Despite its long history in taxonomic research, traditional classification methods based on morphological characteristics have exhibited limitations and uncertainties in both species definition and resolution of phylogenetic relationships [2]. DNA sequence analysis of the semi-autonomous organelles, chloroplasts and mitochondria, which possess their own genomes, has emerged as an indispensable tool in plant taxonomy, augmenting classification precision and advancing phylogenetic research. Compared to the conserved plastid genome, the plant mitochondrial genome (mitogenome) exhibits a relatively low mutation rate but a high frequency of genomic structural rearrangements, making it an important genetic system for studying the evolution of genome structure and functional content [3, 4].

Mitochondria are the central hub of cellular energy metabolism, underpinning the vitality and proliferation of life forms across the eukaryotic domain [5]. Mitochondria originated from α-Proteobacteria that were engulfed by early eukaryotic cells, leading to a symbiotic relationship where the engulfed bacteria provided energy production capabilities, and in return, received protection from the host cell's environment [6, 7]. Mitogenome in most organisms is inherited exclusively from the mother, a pattern known as maternal inheritance [8]. This mode of inheritance simplifies genetic studies by providing a clear lineage of transmission, which is why mitogenome is often utilized in phylogenetic and evolutionary research [9]. Plant mitogenome, compared to plant chloroplast genome and animal mitochondrial genome, varies tremendously in size among different species, spanning a 170-fold range from ~ 66 Kb in *Viscum scurruloideum* to 11,300 Kb in *Silene conica* (Caryophyllaceae) [10–13]. Plant mitogenome exhibit a high degree of plasticity, with genome architecture subject to frequent changes such as gene loss, transfer of genetic material to the nucleus, and various structural mutations and rearrangements [14, 15].

The morphology and organization of mitochondria are much more diverse and dynamic than previously thought. Their structure can range from circular to linear or even branched forms, enriched with repetitive sequences and introns, and they can also be organized into single or multipartite chromosomes [16–20]. The intricate nature of plant mitogenomes often arises from intracellular gene transfer (IGT) and mitogenome recombination events, which are facilitated by extensive repeat sequences. These transfers commonly take place between the chloroplast genome and mitogenome, as well as between the mitochondrial and nuclear genomes. The cumulative effect of such IGT contributes to the enhanced complexity observed in plant mitogenome. Extensive studies on the sequence and structure of plant mitogenome can reveal the primary mechanism, underlying the replication of plant mitogenome, offering genetic diversity, species evolutionary histories, and adaptive mechanisms.

*Cinnamomum longepaniculatum* (Gamble) N. Chao ex H. W. Li (*C. longepaniculatum*), a unique and valuable natural spice tree species belonging to the family Lauraceae, is primarily found in the Yibin region of Sichuan Province, China [21, 22]. *C. longepaniculatum* possesses an exceptionally strong aroma and contains rich concentration of natural essential oils extracted from its leaves and twigs, which exhibit significant applications in the spices, pharmaceuticals, and perfume production [23]. Furthermore, *C. longepaniculatum* plays a pivotal role in ecology, with its robust environmental adaptability and ecological service functions, such as soil improvement and air purification, contributing immeasurably to maintaining regional ecological balance [24]. Consequently, the significance of *C. longepaniculatum* extends beyond its potential economic value, encompassing its crucial role in preserving biodiversity and supporting ecosystem functions.

The emergence of high-throughput sequencing technology has greatly advanced the study of mitogenome, enabling the characteristics of mitogenome in non-model, understudied eukaryotes to be revealed. It has also facilitated the sequencing of large, fragmented, or repetitive-rich mitochondrial genomes, which were difficult to achieve with traditional cloning, standard/long polymerase chain reaction (PCR), and sequencing methods [25, 26].

Although the mitogenome of several species have been reported [27–29], the mitochondrial genomes of family Lauraceae are still poorly studied, severely impeding the in-depth understanding of mitochondrial level biological characteristics. In this study, the assembly of the mitochondrial genome of *C. longepaniculatum* was conducted, and comparative genomics analysis was performed to examine their structural stability and sequence variations. The insights obtained are pivotal for understanding the evolutionary pathway and biochemical attributes of Laurales plants.

## Materials and methods

### Plant materials, DNA extraction, sequencing of mitochondrial genome

The leaves of *C. longepaniculatum* for mitogenome determination were collected from the germplasm resource nursery with elite mother trees of Hongyan Mountain, Yibin, Sichuan Province (28°44′N, 104°48′E), China. In April 2024, six randomly selected healthy *C. longepaniculatum* mother trees aged 20 years were chosen. From each tree, a total of 12 mature leaves were collected in four different directions (east, west, north, and south), with three leaves obtained from the upper, middle, and lower parts of the crown. After being collected, these leaves were thoroughly mixed together, rapidly frozen in liquid nitrogen, and subsequently stored in an ultra-low temperature refrigerator at −80 °C for total DNA analysis.

Both the Illumina Novaseq6000 platform (Illumina, San Diego, CA, USA) and Nanopore PromethION sequencing platform (Nanopore, Oxford, UK) were utilized for the DNA sequencing of *C. longepaniculatum*. The genomic DNA for Illumina was extracted using the plant genomic DNA kit (Tiangen Biotech, Beijing, China), while the samples for PromethION were extracted using the CTAB procedure. The quality and concentration of DNA were assessed through 1% agarose gel electrophoresis, One drop spectrophotometer, and Tanon-1600 Fluorometer.

The high-quality clean reads, totaling 60.83 Mb, were obtained by applying the fastp software [30] to filter the raw Illumina sequencing data. For PromethION sequencing, a total of 4.95 Mb high-quality clean reads were obtained after filtering by filtlong (v0.2.1, https://github.com/rrwick/Filtlong) software.

### Assembly and annotation of *C. longepaniculatum*

The PromethION data were aligned to the reference gene sequence of plant mitochondrial core genes (https://github.com/xul962464/plant_mt_ref_gene) using the minimap2 (v2.1) [31]to obtain the mitogenome sequence. Core genes are highly conserved genes in the mitochondrial genome of most eukaryotes and are usually involved in the core functions of mitochondria (such as oxidative phosphorylation, energy metabolism). These genes are highly homologous between species and are the focus of mitochondrial genome research. Variable genes refer to genes that are present exclusively in specific taxa. These genes exhibit functional diversity and are generally less conserved, potentially reflecting the adaptive evolution of species [32, 33].

Subsequently, the assembly software canu (v2.2) [34] was employed for correcting the obtained PromethION data, while bowtie2 (v2.3.5.1) [35] was utilized for aligning the Illumina data with the corrected sequence. Following this step, Unicycler (v0.4.8) [36], employing default parameters, was used to assemble both the aligned Illumina and corrected PromethION datasets together. Bandage (v0.8.1) software [37] facilitated visualization of assembly results and allowed for manual adjustments.

Mitochondrial genes of *C. longepaniculatum* were annotated by GeSeq [38] software using *Cinnamomum camphora* (*C. camphora*) (NC_086632.1), *Cinnamomum chekiangense* (*C. chekiangense*) (NC_082065.1) and *Caryodaphnopsis henryi*(NC_088584.1) as reference sequences. Default settings of tRNAscan-SE [39] were used to accurately annotated tRNAs. All obtained results underwent thorough review and manual correction prior to final annotation. Visualization of the *C. longepaniculatum* mitogenome was carried out using the Organellar Genome Draw (OGDRAW) (v1.3.1) software [40].

### Codon usage analysis

Due to codon concatenation, each amino acid corresponds to a minimum of one codon and a maximum of six codons. There exists considerable variation in the rate of codon usage across the genomes of different species and organisms. This disparity in synonymous codon usage is referred to as Relative Synonymous Codon Usage (RSCU). The numerical value is the ratio of the actual frequency of codon usage to the theoretical frequency of codon usage. A script written in Perl was used to filter Uniq CDS (http://cloud.genepioneer.com:9929/#/tool/alltool/detail/214) (choosing one of multiple copies of CDS) and do the calculations.

### Repeat sequence analysis

Repeat sequences, encompassing simple sequence repeats (SSRs), tandem repeats, and dispersed repeats were identified. The SSRs were identified utilizing MISA software (v1.0) [41]. Tandem repeats were recognized through the application of TRF software (v4.09), which was executed with a defined set of parameters [42]. Dispersed repeats were discovered by employing BLASTN software (v2.10.1), operating with a specified range of parameters that included the removal of redundancy and crosstalk duplicates. Subsequently, the visualization of these identified repeat sequences was facilitated by Circos (v0.69–5) software [43].

### Homologous sequence analysis

Homologous sequences between chloroplast genome and mitogenome were identified using BLAST software [44]. The E-value was set to 1e-5, while other parameters were kept at their default settings.

### Phylogenetic analysis

Sequences from different species were compared with multiple sequences using MAFFT software (v7) [45]. The aligned sequences were subsequently concatenated, trimmed with TrimAl (v1.4) using the parameter -gt 0.7, and model selection was performed via jModelTest (v2.1.10) [46] to confirm that the appropriate model was of GTR type. Following this, a Maximum likelihood (ML) phylogenetic tree was constructed utilizing RAxML software (v8.2.10) [47], employing the GTRGAMMA model and conducting 1000 bootstrap replicates to assess the stability of the resulting tree.

### RNA editing site prediction of *C. longepaniculatum*

RNA editing site predictions were performed using PmtREP tools available at (http://cloud.genepioneer.com:9929/#/tool/alltool/detail/336) [48]. Based on multiple sequence alignments, the amino acid sequence of the target sequence was compared with multiple sequences in the database. Variations occurring at corresponding positions of the target sequence across the database are then tallied. If the conditions for RNA editing are met, the site is considered a potential RNA editing site.

### Collinearity analysis

The assembled *C. longepaniculatum* mitogenome sequences were compared with those of six closely related species using nucmer software (v4.0.0) [49] for genomic comparisons. Dot-plot plots were generated by utilizing the –maxmatch parameter.

## Results

### Genome features of the mitogenome of *C. longepaniculatum*

**Fig. 1:**
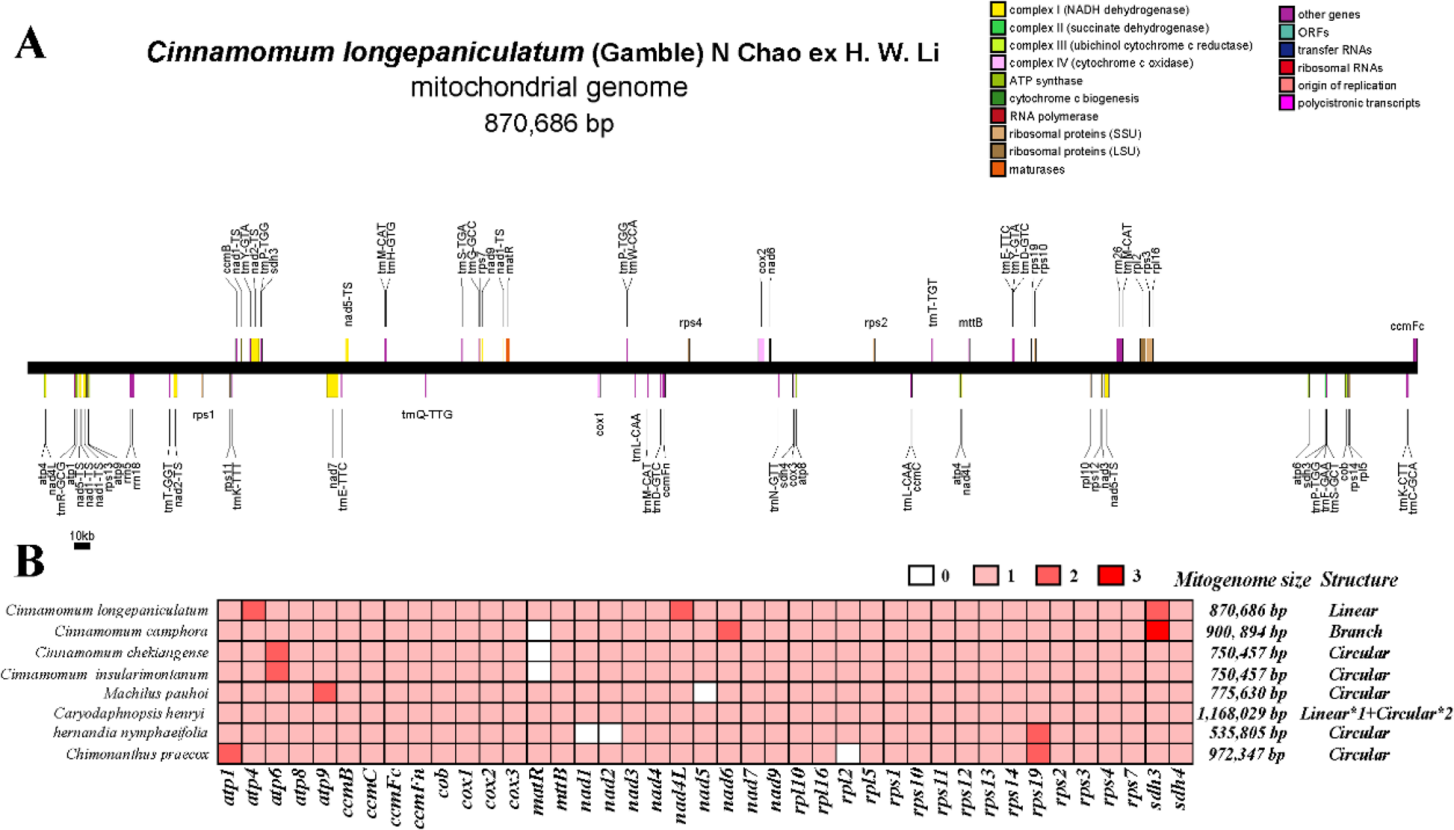
Mitogenome map of* C. longepaniculatum* and PCGs composition of Laurales mitogenomes. **A** Mitogenome map of *C. longepaniculatum*. **B** The composition of PCGs in the mitogenomes of eight Laurales species. White square represents absent genes and varying shades of red blocks reflects different gene copy numbers

The mitogenome of *C. longepaniculatum* was successfully assembled in a computationally efficient manner, employing a unique linear model to encapsulate the entire mitogenome sequence based on long-reads data obtained from PromethION sequencing. The total length of the mitogenome was 870,686 bp, with a GC content of 46.94% (Fig. 1A). This mitogenome size is shorter than that of* C. camphora*, *Caryodaphnopsis henryi*, and *Chimonanthus praecox*, yet longer than observed in four other species within the Laurales (Fig. 1B).

A total of 75 genes were annotated in the *C. longepaniculatum* mitogenome, comprising 44 protein-coding genes (PCGs), which included 26 core mitochondrial genes and 18 non-core genes (Table S1) [50]. Based on their functional characteristics, the PCGs were categorized into ten categories: ATP synthase genes (6), Cytochrome C biogenesis (4), Ubichinol cytochrome C reductase (1), Cytochrome C oxidase (3), Maturases (1), Transport membrance protein (1), NADH dehydrogenase (10), Ribosomal proteins (LSU) (4), Ribosomal proteins (SSU) (11), Succinate dehydrogenase (3) (Table 1). Additionally, 28 tRNA and 3 rRNA (*rrn18*, *rrn26*, *rrn5*) genes were also annotated in *C. longepaniculatum* mitogenome, with 6 tRNAs identified as multicopy (Table 1). Notably, within the mitogenome of *C. longepaniculatum*, a total of 13 genes were found to contain introns. Specifically, the genes *ccmFc*, *cox2*, *rpl2*, *rps1*, *rps2*, *rps3*, *rps10* and *trnT-TGT* each contained one intron. The gene *nad4* contained three introns, while *nad1*, *nad2*, *nad5* and *nad7* each possessed four introns (Table 1).

**Table 1:** Gene organization in the mitogenome of *C. longepaniculatum*

	Group of genes^#^	**Gene names**
**PCGs-Core genes**	ATP synthase	*atp1, atp4(2), atp6, atp8, atp9*
	Cytohrome c biogenesis	ccmB, ccmC, ccmFc*, ccmFn
	Ubichinol cytochrome c reductase	*cob*
	Cytochrome c oxidase	cox1, cox2*, cox3
	Maturases	*matR*
	Transport membrance protein	*mttB*
	NADH dehydrogenase	nad1****, nad2****, nad3, nad4***, nad4L(2), nad5****, nad6, nad7**** nad9
PCGs-Variable genes	Ribosomal proteins (LSU)	rpl10, rpl16, rpl2*, rpl5
	Ribosomal proteins (SSU)	rps1*, rps10*, rps11, rps12, rps13, rps14, rps19, rps2*, rps3*, rps4, rps7
	Succinate dehydrogenase	*sdh3(2), sdh4*
rRNAs	Ribosomal RNAs	*rrn18, rrn26, rrn5*
tRNAs	Transfer RNAs	trnC-GCA, trnD-GTC(2), trnE-TTC(2), trnF-GAA, trnG-GCC, trnH-GTG, trnK-CTT, trnK-TTT, trnL-CAA(2), trnM-CAT(3), trnN-GTT, trnP-TGG(3), trnQ-TTG, trnR-GCG, trnS-GCT, trnS-TGA, trnT-GGT, trnT-TGT*, trnW-CCA, trnY-GTA(2)
	other	

Note: ^*, ***, ****^ represent the total count of introns; # indicates the Pseudo gene; (2) (3) represent the copy number of multi-copy genes

**Fig. 2:**
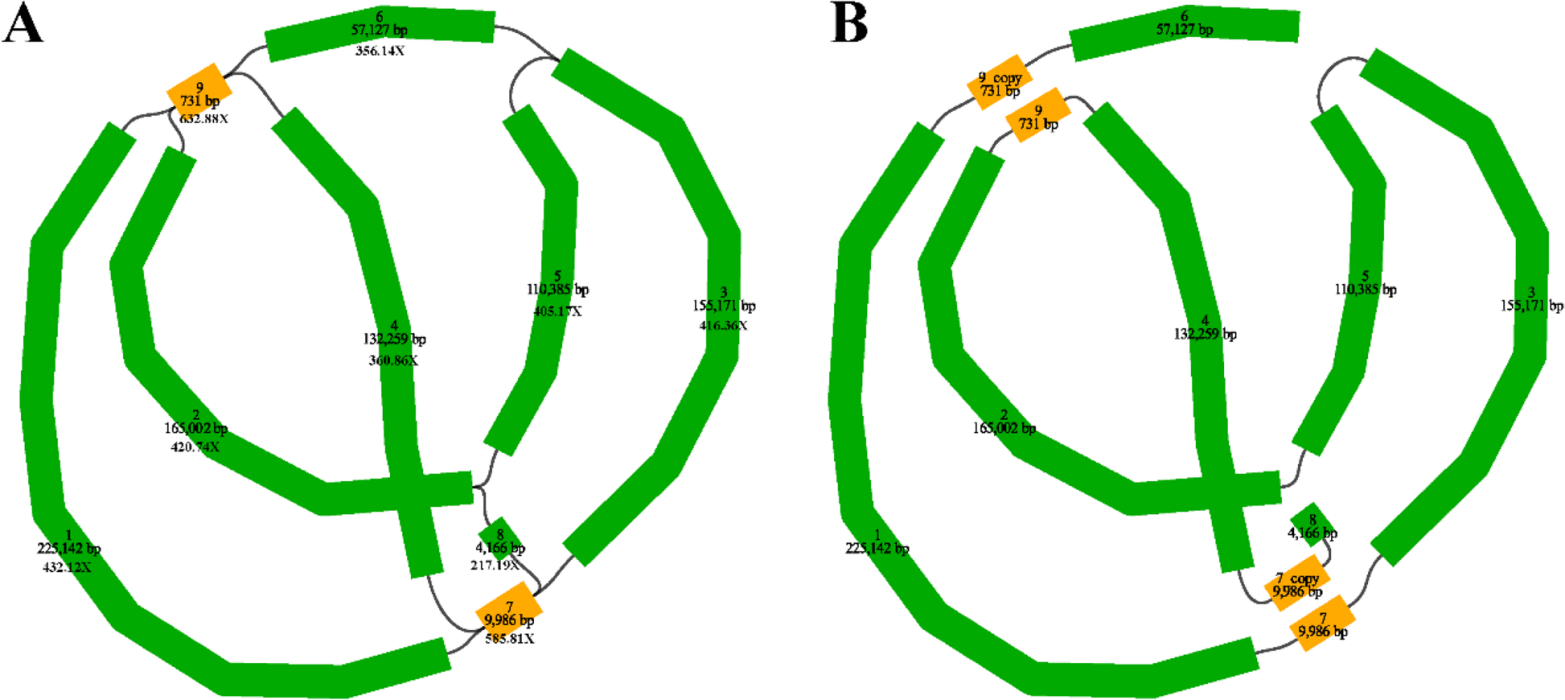
**A** Schematic of *C. longepaniculatum* mitogenome based on Flye assembly. **B** Unicycler hybrid assembly-based mitogenome of *C. longepaniculatum* (node ID labeled in the figure). The mitogenome schematic was generated using Bandage software based on PromethION data. Each contig represented a sequence obtained from the assembly process. Contigs connected by a black line indicated an overlapping region between them

The schematic comprised nine contigs, with its name, sequence length, and sequencing depth as provided in Fig. 2A. The nine contigs formed a complex and branched structure, representing the complete mitogenome of *C. longepaniculatum*. Contig 7 and contig 9 were two sequences of key nodes. The resulting genome sequence followed the path 6-9_copy-1–7-3–5-2–9-4-7_copy-8 (Fig. 2B). It is important to note that this method was not the exclusive form due to the dynamic nature of plant mitogenome structures. However, it was chosen to facilitate subsequent analysis.

### Codon usage preference of PCGs

The cumulative length of PCGs in *C. longepaniculatum* amounted to 35,367 bp, accounting for 4.06% of the whole mitogenome (Table S2a). In the *C. longepaniculatum* mitogenome, a total of 11,378 codons were utilized to encode 44 PCGs. All annotated PCGs initiated with the ATG codon and exhibited three types of termination codons (TAA, TGA, and TAG), with frequencies of utilization at 45.45%, 34.09%, and 20.45%, respectively (Table 2). The three most frequently employed amino acids were Ser (9.78%), Leu (9.44%), and Ile (7.49%), whereas Ter was the least frequent, occurring only 41 times (0.36%) (Table 2). Further analysis revealed 31 codons were found to be more prevalent (RSCU > 1), while 30 codons were less frequently employed (RSCU < 1). Notably, the codons for Met (AUG), Ser (AGU) and Trp (UGG) exhibited RSCU values of 1, indicating their standard levels of utilization. Among these high-frequency codons (RSCU > 1), 28 ended in either A or U, accounting for a significant proportion of 90.3%. This indicated a strong inclination towards codons ending in NNA or NNU in the mitogenome of *C. longepaniculatum* (Table 2).

**Table 2:** Codon counts and RSCU in *C. longepaniculatum* mitochondrial PCGs

Amino Acid	Symbol	Codon	Count	RSCU	Amino Acid	Symbol	Codon	Count	RSCU
*	Ter	UAA	19	1.39	M	Met	AUG	311	1.00
*	Ter	UGA	13	0.95	N	Asn	AAU	248	1.36
*	Ter	UAG	9	0.66	N	Asn	AAC	117	0.64
A	Ala	GCU	288	1.58	P	Pro	CCU	240	1.35
A	Ala	GCA	184	1.01	P	Pro	CCA	202	1.14
A	Ala	GCC	158	0.87	P	Pro	CCC	148	0.83
A	Ala	GCG	98	0.54	P	Pro	CCG	121	0.68
C	Cys	UGU	93	1.16	Q	Gln	CAA	240	1.49
C	Cys	UGC	68	0.84	Q	Gln	CAG	82	0.51
D	Asp	GAU	261	1.41	R	Arg	AGA	184	1.33
D	Asp	GAC	110	0.59	R	Arg	CGA	180	1.30
E	Glu	GAA	316	1.32	R	Arg	CGU	170	1.22
E	Glu	GAG	162	0.68	R	Arg	CGG	108	0.78
F	Phe	UUU	345	1.06	R	Arg	AGG	102	0.73
F	Phe	UUC	307	0.94	R	Arg	CGC	89	0.64
G	Gly	GGA	294	1.44	S	Ser	UCU	242	1.30
G	Gly	GGU	256	1.26	S	Ser	UCA	214	1.15
G	Gly	GGG	152	0.75	S	Ser	UCC	188	1.01
G	Gly	GGC	112	0.55	S	Ser	AGU	186	1.00
H	His	CAU	229	1.54	S	Ser	UCG	171	0.92
H	His	CAC	68	0.46	S	Ser	AGC	112	0.60
I	Ile	AUU	365	1.29	T	Thr	ACU	198	1.36
I	Ile	AUC	256	0.90	T	Thr	ACC	153	1.05
I	Ile	AUA	231	0.81	T	Thr	ACA	147	1.01
K	Lys	AAA	295	1.19	T	Thr	ACG	86	0.59
K	Lys	AAG	202	0.81	V	Val	GUA	211	1.20
L	Leu	UUA	237	1.32	V	Val	GUU	194	1.10
L	Leu	CUU	231	1.29	V	Val	GUG	172	0.98
L	Leu	UUG	223	1.25	V	Val	GUC	128	0.73
L	Leu	CUA	155	0.87	W	Trp	UGG	153	1.00
L	Leu	CUC	123	0.69	Y	Tyr	UAU	236	1.49
L	Leu	CUG	105	0.59	Y	Tyr	UAC	80	0.51

Note: The asterisk (*) denotes a stop codon

**Fig. 3:**
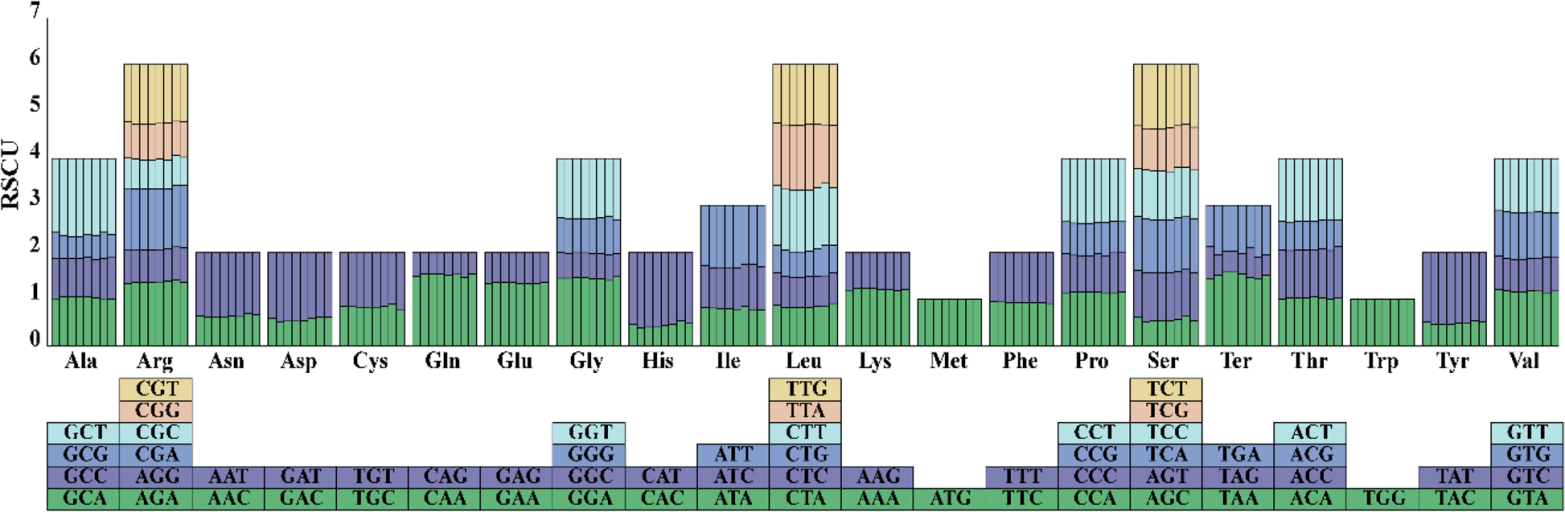
Relative synonymous codon usage (RSCU) of PCGs within eight species of Laurales. The x-axis represents codon families. The stacked bar graph for each codon from left to right is the species of *C. longepaniculatum, C. camphora, C. chekiangense, C. insularimontanum, Machilus pauhoi, Caryodaphnopsis henryi, Hernandia nymphaeifolia* and *Chimonanthua praecox.* RSCU values represents specific codon frequency in comparison to uniform synonymous codon usage expected frequency

In the eight complete mitogenome sequences of Laurales plants, a total of 64 different types of codons encoding all 20 amino acids were also identified. The three most frequently utilized amino acids were also Ser, Leu, and Ile, with total count of more than 1066, 1090 and 795, respectively (Table S2b). Among the mitochondrial PCGs of *C. longepaniculatum*, alanine (Ala) exhibited a distinct preference for GCU with highest RSCU value of 1.58. Following closely was histidine (His), which favored CAU with an RSCU value of 1.54 (Table 2). Similarly, in the seven species of Laurales plants, the preferred codons for alanine and histidine were also GCU and CAU, respectively, which demonstrates a strongly conserved preference across Laurales species (Table S2b, Fig. 3).

### Repeat sequences analysis

**Fig. 4:**
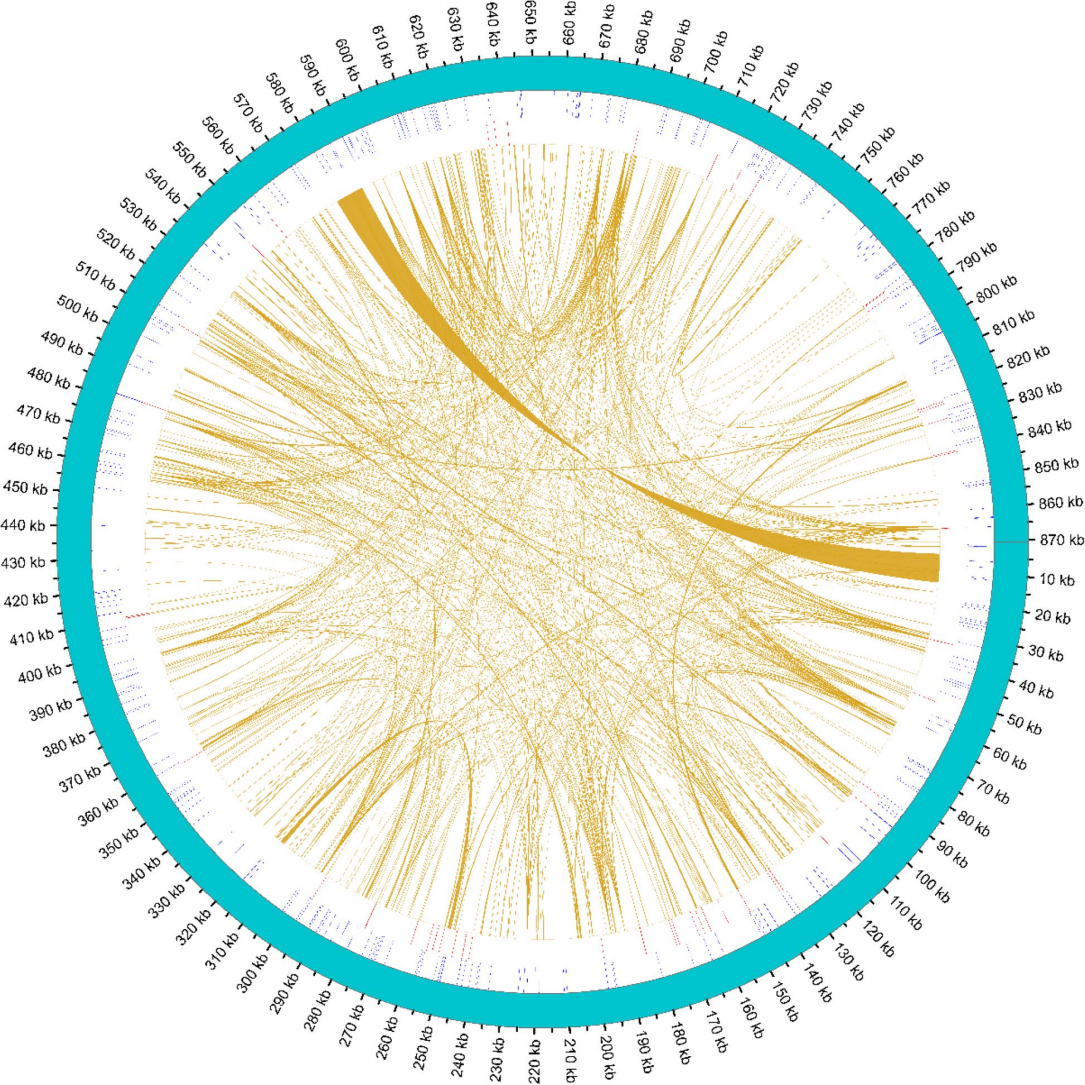
Distribution of repetitive sequences within mitogenome of *C. longepaniculatum*. The outermost circle represented the mitogenome sequence, followed inward by simple sequence repeats, tandem repeated sequences, and dispersed repeated sequences

**Fig. 5:**
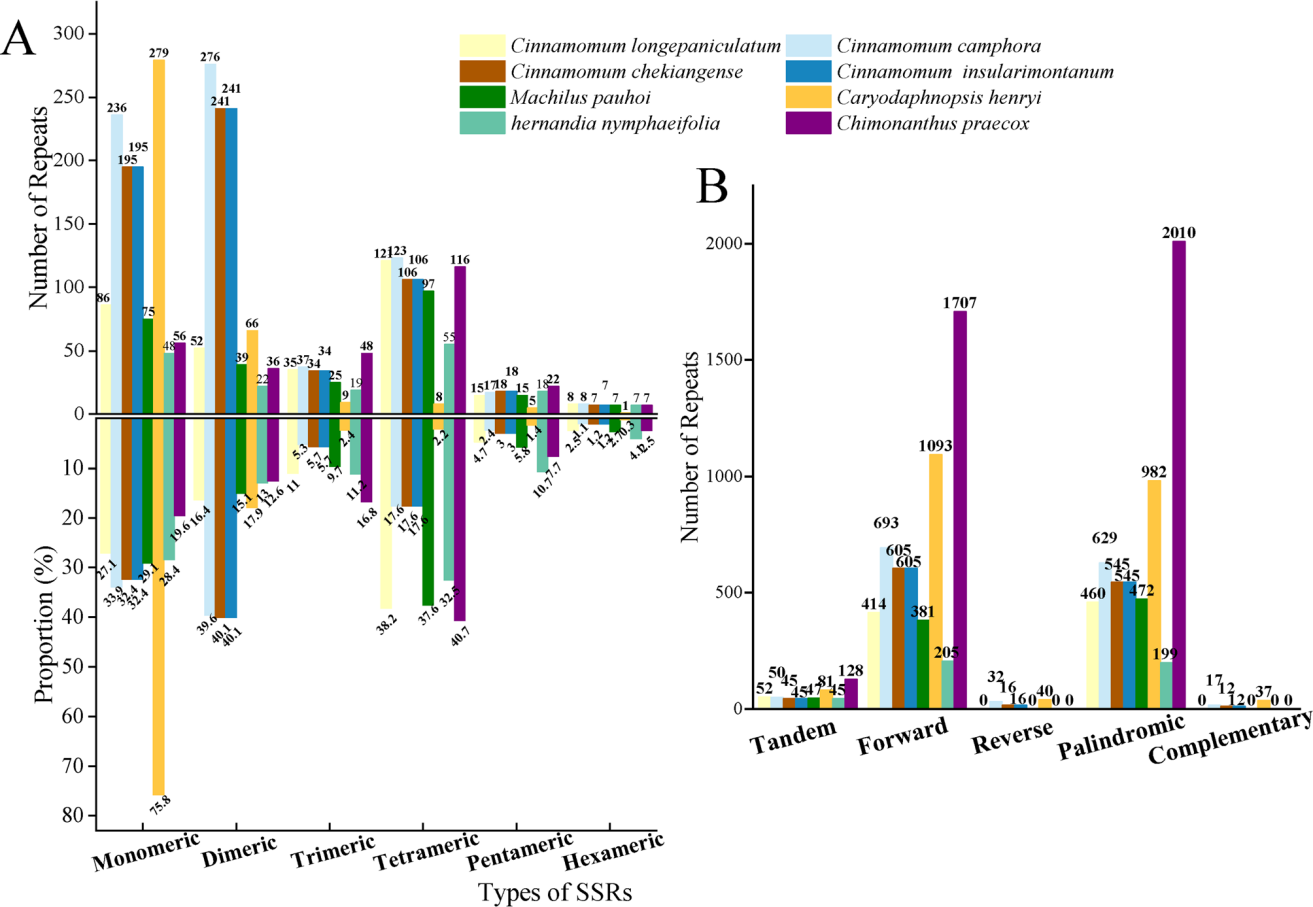
Histogram of repetitive sequence analysis. **A** SSRs Types in eight Laurales species. The horizontal axis represented SSR types, while the vertical axis displayed the number of repetitive fragments. **B** Tandem and dispersed repeats in eight Laurales species. The horizontal axis represented the types of repeats, while the vertical axis illustrated the number of repeats

The repetitive sequence, including simple sequence, tandem, and dispersed repeats, was analyzed in the mitogenome of *C. longepaniculatum* (Fig. 4). A total of 317 SSRs were identified, encompassing repeat types ranging from monomeric to hexameric nucleotides in length. Notably, the monomeric and tetrameric repeats were the predominant types with 86 and 121 repeats, accounting for 27.1% and 38.2%, respectively (Table S3). This pattern of distribution aligned closely with observations in the mitogenomes of *Machilus pauhoi, hernandia nymphaeifolia and Chimonanthus praecox*. However, in the mitogenomes of *C. camphora, C. chekiangense, C. insularimontanum and Caryodaphnopsis henryi*, monomeric and dimeric repeats were more abundant, particularly in *Caryodaphnopsis henryi*, where monomeric repeats constituted as high as 75.8% of the SSRs (Table S3, Fig. 5A).

A total of 52 tandem repeat sequences were identified within the *C. longepaniculatum* mitogenome, with matching degrees exceeding 76% and lengths ranging from 2 to 48 bp. The copy number variation of these 52 tandem repeat sequences ranged between 1.9 and 16.2, among them, three nucleotide sequences had copy numbers exceeding 10, specifically the dinucleotide repeat sequences AT and TA, and the tetranucleotide repeat sequence AAAG (Table S4).

The REPuter software also detected dispersed repeats in the *C. longepaniculatum* mitogenome [51], revealing a significant number of dispersed repeats totaling 874 pairs, with lengths equal to or greater than 30 bp. Among these, there were 414 pairs of forward repeats (F) and 460 pairs of palindromic repeats (P), without any reverse or complementary repeats (Table S5). The longest palindromic repeat was found to be 958 bp in length, while the longest forward repeat spanned an impressive length of 9991 bp (Table S6). Additionally, an analysis of the mitogenomes of seven other Laurales species revealed that *Machilus pauhoi* exhibited a similar number of forward repeats (381) and palindromic repeats (472) as compared to *C. longepaniculatum* (Table S5). Particularly, our analysis of *C. longepaniculatum* mitogenome did not reveal any reverse or complementary repeats, a finding that was consistent with observations in mitogenomes of *Machilus pauhoi, hernandia nymphaeifolia and Chimonanthus praecox* (Fig. 5B).

### Homologous analysis of genome sequences

**Fig. 6:**
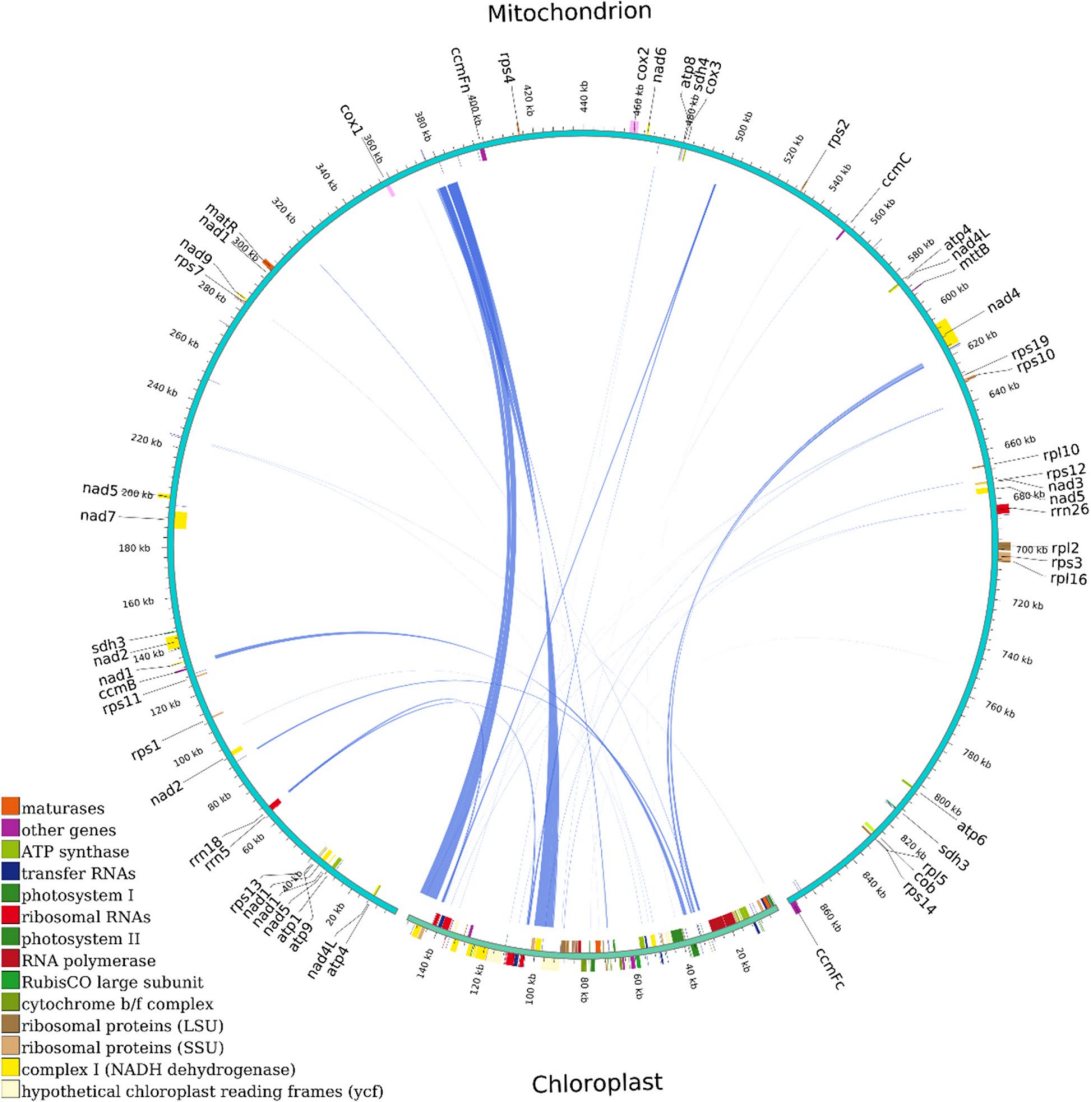
Homology analysis of the mitochondrial and chloroplast genomes of *C. longepaniculatum*. The upper arcs symbolized the mitogenome, the lower arcs denoted the chloroplast genome, and blue lines connecting the arcs indicated homologous sequences. Blocks of the same color symbolized genes from the same complex, with outer and inner circles indicating genes on the positive and negative strands, respectively

The chloroplast genome of *C. longepaniculatum* exhibited a length of 152,730 bp, encompassing 129 genes (Table S7). Through sequence similarity analysis, a total of 35 fragments were homologous sequences to the chloroplast genome and mitogenome, with more than 75.8% similarity. The length of these fragments ranged from 32 to 4,741 bp, with six fragments exceeding 1000 bp (Table S8). These homologous sequences had a cumulative length of 15,462 bp, constituting approximately 1.78% of the overall mitogenome length (Fig. 6). By annotating these homologous sequences, a total of 10 tRNA genes (*trnH-GTG*, *trnL-CAA*, *trnN-GTT*, *trnT-GGT*, *trnM-CAT*, *trnE-TTC*, *trnY-GTA*, *trnP-TGG*, *trnW-CCA*, *trnD-GTC*) in mitogenome were completely located within these homologous sequences, while two rRNA genes (*rrn18*, *rrn26*) were partially located within them. Additionally, some chloroplasts genes were also observed on these homologous sequences, including three PCGs (*rps7*, *ndhB* and *petN*) and eleven tRNA (Table S9).

### Phylogenetic analysis

**Fig. 7:**
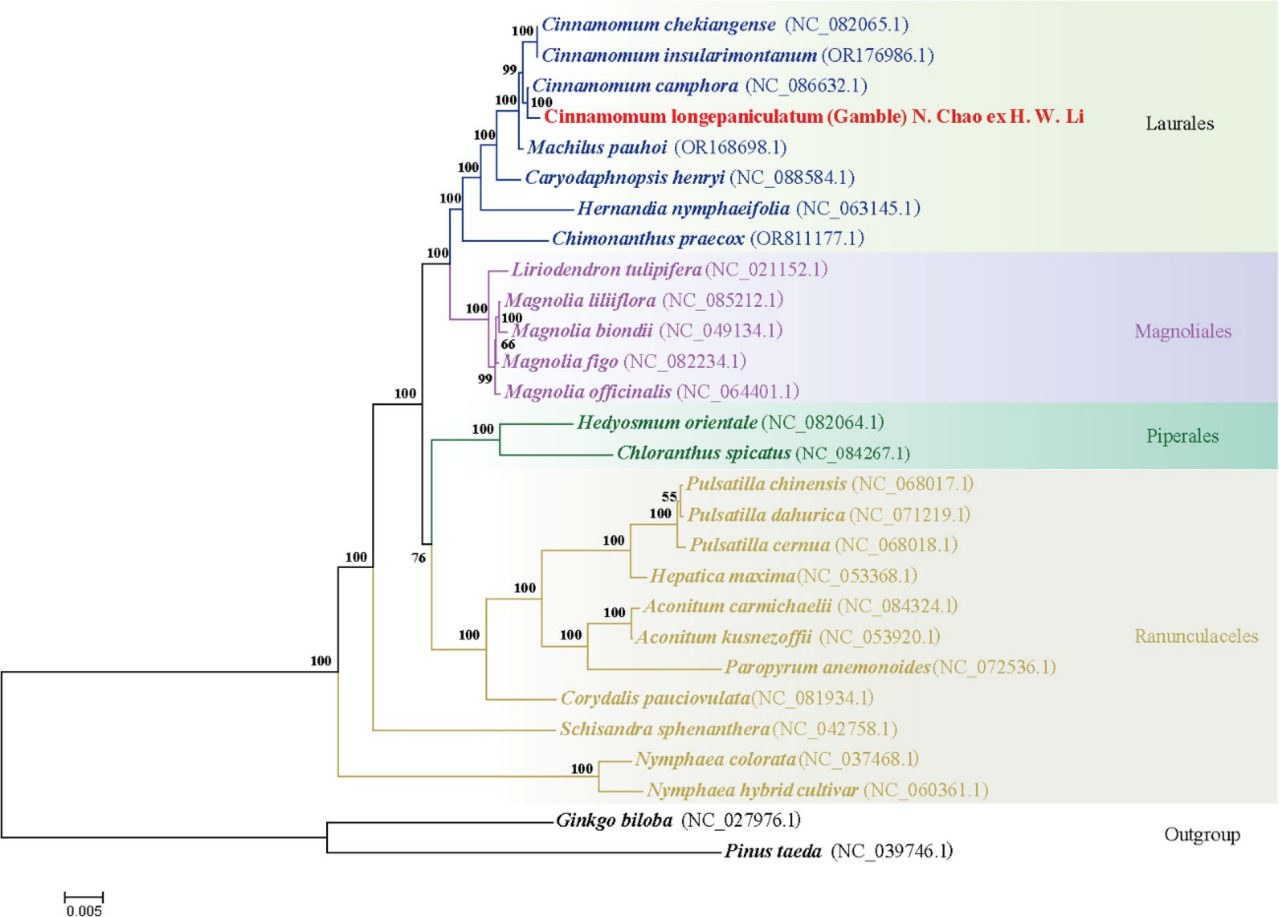
The phylogenetic associations of *C. longepaniculatum*. alongside 28 other plant species. Phylogenetic tree constructed by maximum likelihood method based on 41 conserved mitochondrial genomic PCGs. Numerical values at each node represented bootstrap probability. Different colors indicated the respective orders that the specific species belong. *Ginkgo bilob*a and *Pinus taeda* were used as outgroup species

Based on the DNA sequences of 41 conserved mtPCGs (*atp1*, *atp4*, *atp6*, *atp8*, *atp9*, *ccmB*, *ccmC*, *ccmFc*, *ccmFn*, *cob*, *cox1*, *cox2*, *cox3*, *matR*, *mttB*, *nad1*, *nad2*, *nad3*, *nad4*, *nad4L*, *nad5*, *nad6*, *nad7*, *nad9*, *rpl10*, *rpl16*, *rpl2*, *rpl5*, *rps1*, *rps10*, *rps11*, *rps12*, *rps13*, *rps14*, *rps19*, *rps2*, *rps3*, *rps4*, *rps7*, *sdh3*, *sdh4*), a phylogenetic tree was constructed for 26 species belonging to four orders of magnoliids. In the resulting phylogenetic tree analysis, 22 out of 25 branching nodes exhibited bootstrap values exceeding 99%, with 20 nodes having a bootstrap value of 100%. The topological arrangement of the phylogenetic tree aligned consistently with the latest identification by the Angiosperm Phylogeny Group (APG) when rooted in mitogenome sequences. It was worth noting that the *C. longepaniculatum* was classified within the family Lauraceae of the Laurales order and demonstrated a close phylogenetic relationship with *C. camphora,* followed by the *C. chekiangense* and the *C. insularimontanum* species (Fig. 7).

### Prediction of RNA editing sites

**Fig. 8:**
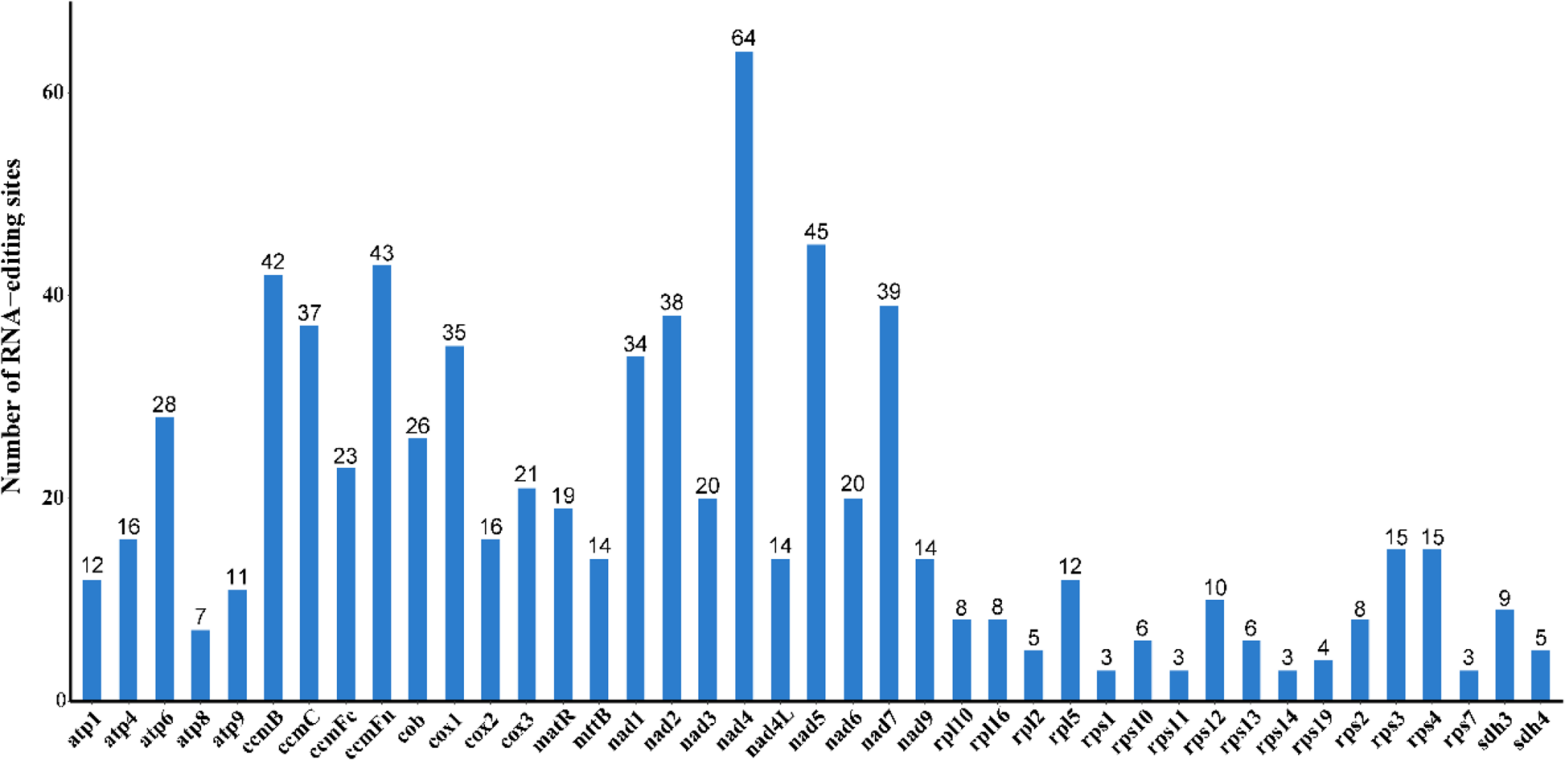
Illustration of RNA editing site distribution across individual mitochondrial PCGs in *C. longepaniculatum*. The blue bars depict the number of RNA-editing sites for each gene

The PmtREP software was used to predict RNA editing events in 44 unique PCGs from *C. longepaniculatum* mitogenome. A total of 761 potential RNA editing sites were identified, characterized by consistent C to T conversions. The RNA editing sites identified involve changes in 25 codons, which mainly at the second base (n = 491, 64.52%), followed by the first base (n = 240, 31.54%), with the both changes occurring at the first and second base (n = 30, 3.94%) (Table S10). Notably, the *nad4* gene exhibited the highest frequency of RNA editing sites with a total of 64 edits, while *rps1*, *rps7*, *rps11* and *rps14* had only 3 editing site each. The number of change sites in the remaining genes ranged from 4 to 45 (Fig. 8). In addition, there were a total of 31 codon variations implicated in RNA editing sites. Among them, 43.76% of amino acids showed no alteration in hydrophobicity. Conversely, 46.52% were predicted to transition from a hydrophilic to a hydrophobic state, while 8.94% were anticipated to shift from hydrophobic to hydrophilic (Table S11). Furthermore, it was found that RNA editing events could potentially introduce stop codons in the *atp6*, *atp9*, *rps10*, *rps11*, *rpl16* and *ccmFc* genes (Table S10. Table S11).

### Collinearity analysis

**Fig. 9:**
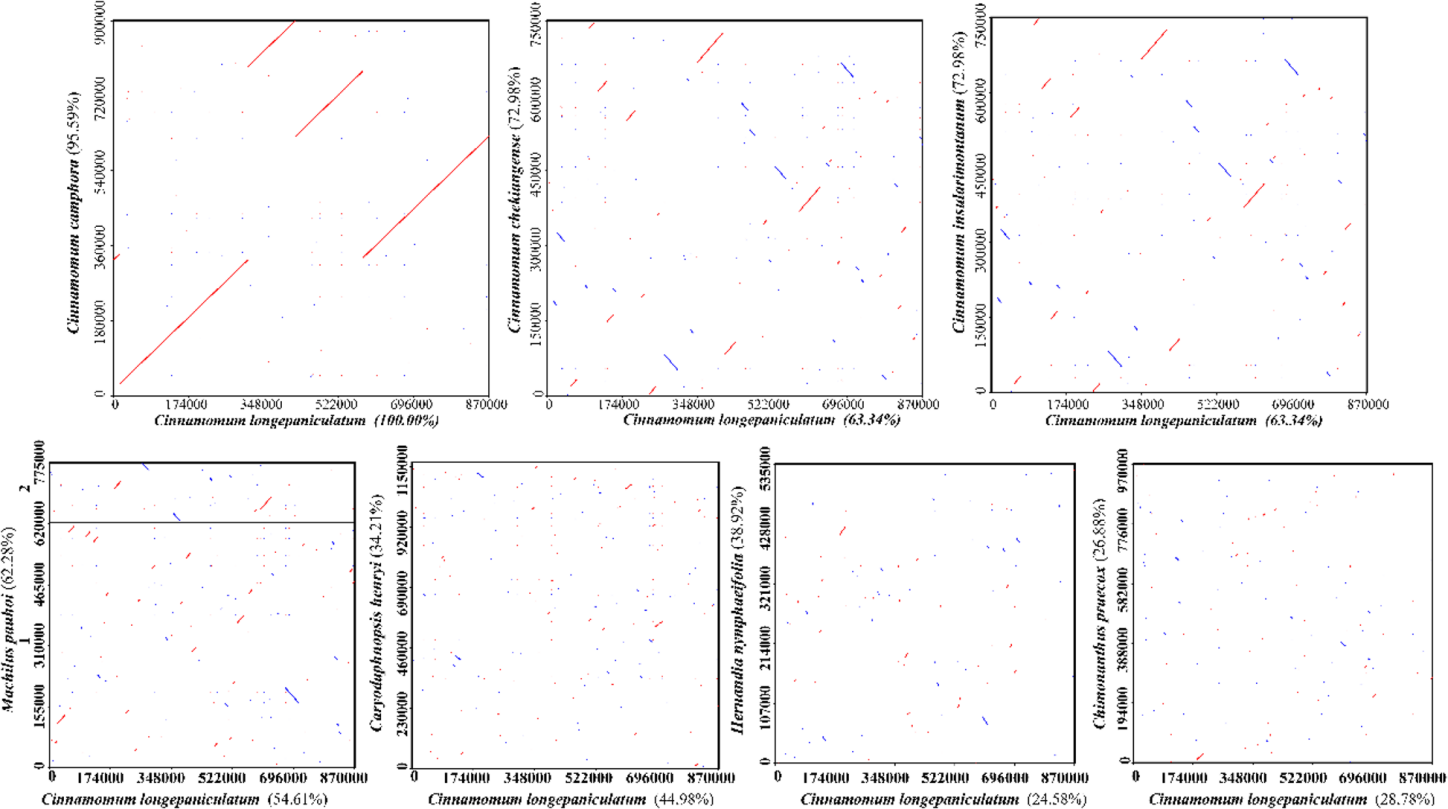
Dot-plots analysis. The sequence of *C. longepaniculatum* were plotted along the horizontal axis in each box, while the sequences of the other seven species were represented along the vertical axis. The numbers in parentheses indicated the proportion of homologous sequences in relation to the total genome. The red lines in the box denoted forward alignment, whereas the blue lines represented reverse complementary alignment

The dot-plot analysis revealed a substantial number of variations in collinearity among the mitogenomes of *C. longepaniculatum* and seven related species within the Laurales order. Notably, the largest co-linear blocks, spanning 861,135 bp, were observed in comparison with *C. camphora*, accounting for approximately 95.59% of the total proportion, followed by *C. chekiangense* and *C. insularimontanum* with 72.98% (Table S12). These findings indicate that *C. longepaniculatum* shared a high degree of genetic similarity and evolutionary conservation with species within the Lauraceae family, particularly with *C. camphora*. Conversely, the collinearity between *C. longepaniculatum* and species from other families in the order Laurales is markedly lower, with only 38.92% in *Hernandia nymphaeifolia* (Hernandiaceae) and 26.88% in *Chimonanthus praecox* (Calycanthaceae) (Fig. 9). These results suggest greater divergence in mitogenome structure and evolution across these lineages.

## Discussion

### Structural characteristics of the *C. longepaniculatum* mitogenome

Plant mitogenome exhibits a higher level of complexity compared to those of animals, characterized by substantial variations in size, intricate sequence arrangements, abundant repetitive content, and a remarkably conserved coding sequence [13, 52, 53]. Deciphering the architecture of these mitogenomes is crucial for elucidating their functionalities, replication mechanisms, modes of inheritance, as well as their evolutionary pathways. Although the plant mitogenomes are frequently assembled and depicted in the form of circular maps, it is most probably not a single, large, circular DNA molecule. Instead, it primarily exists as an intricate and ever-changing assortment of linear DNA segments, along with a mix of smaller, circular, and branched DNA molecules [54–56]. The mitogenome of order Laurales that has been identified exhibits complex and varied structures, which included branched (*C. camphora*) [27], one large linear plus two small circular structures (*Caryodaphnopsis henryi*) [28], single circular (*C. chekiangense*) [29], and double circular structure (*Machilus pauhoi*) [57]. The mitogenome of our *C. longepaniculatum* displayed a linear arrangement and consisted of 9 contigs with a total genome size of 870,686 bp, which added another dimension to this structural repertoire (Fig. 1, Table 1).

Despite these structural variations, *C. longepaniculatum* shares core PCGs essential for oxidative phosphorylation (e.g., *atp1*, *cox1*, *nad2*) with other Laurales species, probably highlighting the conservation of fundamental energy-production mechanisms in mitogenomes of this family. Notably, 24 core PCGs were all identified in *C. longepaniculatum* mitogenome, which were inconsistent with the identification results of missing *matR* core genes in *C. camphora*, *C. chekiangense and C. insularimontanum.* This indicates that the core PCGs sequence in mitogenome of *C. longepaniculatum* are not lost during mitochondrial symbiosis. The specific function and related mechanism of this mitochondrial gene still need to be further explored and verified.

### Codon usage analysis of PCGs

The eukaryotic genome comprises 64 codons, with substantial variation in the frequency of codon utilization among different species. This preference is thought to be the result of a relative equilibrium that gradually develops within the cell over a long period of evolutionary selection [58]. The PCGs in the mitogenome of *C. longepaniculatum* accounted for only 4.06% (35,367/870,686) of the total sequence length, similar to 3.59% observed in *Caryodaphnopsis henryi* [47]. This suggests a significant presence of non-coding sequences within the mitogenome, which might contain regulatory elements crucial for replication, transcription, and expression regulation of mitogenome. Alternatively, this low proportion of PCGs could suggest a more streamlined and efficient encoding strategy employed by *C. longepaniculatum* to prioritize the synthesis of essential mitochondrial proteins, while minimizing the metabolic cost associated with mitogenome replication and maintenance [59]. Analysis of codon usage through RSCU unveiled a notable adenine/thymine (A/U) richness (90.3%) at third codon position within *C. longepaniculatum* mitogenome. Similarly, the proportion in the other Laurales species ranged from 90.6% to 93.9% (Table S2). The prevalence of NNA and NNU codons underscores a pronounced bias towards a nucleotide at the third codon. This observed pattern likely results from a combination of mutational pressure and selection for efficient translation within the mitochondria [60]. Further investigation is needed to elucidate the specific functional and regulatory roles of these codon usage patterns in the mitochondrial biology of *C. longepaniculatum.*

The PCGs of eight Laurales plants exhibited a significant preference for the codons encoding Ser, Leu and Ile, while the usage frequency of Ter was relatively low. This phenomenon likely reflects the functional requirements of the mitogenome. Specifically, Leu and Ile participate in energy metabolism through the branched-chain amino acid metabolic pathway, and Ser supports mitochondrial function through the one-carbon metabolic pathway. These processes are crucial for maintaining the normal function of mitochondria [61]. In addition, the low-frequency usage of termination codons may be related to the small size of the mitogenome and the efficient gene expression regulation mechanism to reduce the risk of functional disorders caused by premature termination [62].

### Repeat sequences

Repeated sequences are important sources of information for the development of population and evolutionary analysis markers. Tandem, SSR, and long repeats are widely present in mitochondrial genomes. The repeated sequences in plant mitogenome are critical to intermolecular recombination, which can generate structural variations and extreme mitogenome sizes [63–65]. The detection of 52 tandem repeat sequences in *C. longepaniculatum*, with high matching degrees (more than 76%) and varying copy numbers (from 1.9 to 16.2), indicates a dynamic region of the mitogenome that may be subject to frequent recombination events. The presence of tandem repeats with high copy numbers, such as the dinucleotide repeats AT and TA, and the tetranucleotide repeat AAAG, could have implications for genome stability and gene expression regulation (Table S4). The high A/T composition in SSRs likely contributes to the overall AT richness observed in *C. longepaniculatum* mitogenome. This trend aligns with broader patterns observed across land plants, suggesting a consistent evolutionary trajectory in terms of base composition within plant organelle genomes [66–68].

Interestingly*, C. longepaniculatum* exhibits a similar number of forward and palindromic repeats to *Machilus pauhoi*, further supporting the shared structural features among these closely related species. However, the significantly longer palindromic repeat (958 bp) and forward repeat (9,991 bp) identified in *C. longepaniculatum* highlight its unique genomic complexity. Importantly, these repetitive sequences not only facilitate the expansion of the size of mitogenome but also signify the recurrent and frequent occurrence of intermolecular recombination within mitogenome molecules [39, 69]. Further research into the functional implications of these repeat sequences could provide deeper insights into the molecular mechanisms shaping the evolution of mitochondrial genomes in plants.

### Homology analysis

Angiosperms exhibit substantial exchange of genetic material among organelle genomes, facilitated by the transfer of DNA from chloroplasts to the mitochondrial DNA, a process termed MTPTs (Mitochondrial plastid DNAs) [70]. Monitoring gene migration is crucial for unraveling the evolutionary trajectory of the plant mitogenome. This phenomenon primarily accounts for the variations in the number of coding genes observed across diverse plant species [71]. Based on the data, it was found that 15,462 bp of homologous sequences between the mitogenome and chloroplast genome of *C. longepaniculatum*, representing approximately 1.78% of the total length of the mitogenome. Generally, genes that migrate from chloroplast genome to mitogenome frequently evolve into pseudogenes, losing their functional significance, potentially as a result of sequence recombination events [72, 73]. Previous studies have demonstrated that the two pseudogenes *ψndhB* and *ψcemA* in the *Aeginetia indica* mitogenome were transferred from its plastid genome [74]. These homologous segments vary interspecifically in terms of both their length and sequence composition. In this study, 10 intact tRNA genes were completely located in homologous sequences, accounting for 35.71% (10/28) of the total tRNA in the *C. longepaniculatum* mitogenome. The translocation of tRNA genes from chloroplast genome to mitogenome a represents a recurring pattern in plant evolutionary history. Notably, as plants evolve from simpler to more complex taxonomic groups, the incidence of such tRNA transfers intensifies, paralleling the escalating requirements for protein biosynthesis [75]. The chloroplast genome of *C. longepaniculatum* significantly contributes numerous sequences to the mitogenome, thereby enriching the diversity of its mitogenome. However, the underlying mechanisms that govern the migration of sequences between these genomes and the expression patterns of genes within the transferred sequences remain elusive, necessitating further exploration and research.

### RNA editing sites prediction

RNA editing refers to the phenomenon of insertion, deletion, and substitution of bases in the coding region of RNA after transcription [76]. This is a common phenomenon in the transcriptome, particularly in the transcription process of plant mitochondrial genes, which can lead to great diversity of posttranscriptional gene sequences [77]. Earlier studies have reported 782 RNA editing sites in 40 PCGs of *C. camphora*, 1119 in 41 *C. chekiangense* PCGs [27, 29]. In this study, the 761 RNA editing sites were identified across 44 PCGs, with *nad4* showing the highest frequency of RNA editing (64 sites) (Fig. 8). This finding is consistent with the observation of the highest editing events (68 sites and 77 sites) for the *nad4* gene in *C. camphora* and *C. chekiangense*. The *nad4* gene, encoding a core subunit of Complex I (NADH dehydrogenase) in the electron transport chain, plays a critical role in ATP synthesis and mitochondrial energy production [78]. RNA editing at multiple sites within *nad4* may fine-tune its catalytic efficiency or regulatory interactions, ensuring adaptation to metabolic demands.

The RNA editing sites in *C. longepaniculatum* exclusively exhibited transitions from C to T, mainly occurring at the first (31.54%, 240 sites) and second (64.52%, 491 sites) editing position in codon (Table S10). This positional preference aligns with the observed pattern of RNA editing changes in other angiosperms, where editing at the second base often modulates amino acid hydrophobicity or maintains conserved protein domains [79, 80]. The impact of RNA editing on protein structure is highly significant, as it alters the start and stop codons of PCGs, thereby modifying the placement of the initiation and termination signals for translation [81]. For instance, *cox1*, *nad1*, *nad4L* and *rps10* were found to possess ACG start codons that are corrected to the universal AUG start codon via RNA editing [79]. In *C. camphora,* an ACG-to-AUG editing event in *nad4L* generates the correct start codon [27]. Additionally, in *Asparagus officinalis*, five amino acid coding sites (0.82%) resulted in stop codons, specifically located in the *atp9* and *ccmFc* gene (CGA-TGA), *rps11* and *atp6* gene (CAA-TAA), and *rpl16* gene (CAG-TAG) [54]. The generation of new start and stop codons through RNA editing leads to the production of more evolutionarily conserved proteins, which exhibit high homology with corresponding proteins in other species. This conservation enhances the expression of mitochondrial genes [82].

### Phylogenetic analysis and collinearity

The phylogenetic analysis of mitogenome has gained increased attention to elucidate the intricate evolutionary relationships. In this study, we used 41 conserved PCGs to reconstruct the phylogenetic trees of 26 Magnoliids species. Eight species of Laurales formed a closely related cluster and exhibit a close phylogenetic relationship with Magnoliales, as compared to Piperales and Ranunculaceles. This finding aligns with the results reported by Han et al. concerning *C. camphora* [27] and is consistent with the Angiosperm classification system APG IV [83]. It is worth noting that although plant mitogenome play a key role in plant evolutionary studies and provide unique evolutionary information, the total number of plant mitogenomes that have been completely sequenced is still limited, and constructing a phylogenetic tree solely based on mitogenome may not precisely mirror the accurate phylogenetic relationships. Therefore, the complete DNA information of species can be obtained by whole genome sequencing in the future, and the problem of data bias can be better solved by constructing molecular phylogenetic trees at the gene level [84, 85].

We identified homologous co-linear blocks among *C. longepaniculatum* and other seven species of Laurales in the comprehensive comparative analysis. The dot-plot analysis revealed distinct patterns of genomic conservation and divergence. High collinearity, such as the 95.59% similarity observed between *C. longepaniculatum* and *C. camphora*, was concentrated in dense red forward alignments along the diagonal, suggesting that these two species likely share a relatively recent common ancestor within the Lauraceae family. The low collinearity from other families may reflect differences in genomic conservation and the potential influence of evolutionary processes such as gene rearrangements, insertions, or deletions across these lineages. Such extensive rearrangements are likely drivers of evolution and contributors to the observed mitogenome diversity in this species [20, 86]. Further investigation into the specific mechanisms underlying these rearrangements can provide insights into the molecular basis of evolutionary adaptation and the generation of biodiversity in Laurales species.

## Conclusions

In this study, we completed the assembly and annotation of the entire *C. longepaniculatum* mitogenome. The assembled mitogenome exhibited a linear structure with a length of 870,686 bp and a GC content of 46.94%. A total of 75 genes, comprising 44 PCGs, 28 tRNA genes, and 3 rRNA genes were annotated. Codon usage preference analysis revealed that among the 31 codons with RSCU > 1, there was a strong preference for ending in A/U, highlighting conserved translational optimization strategies. Repeat sequence analysis uncovered 317 SSRs and abundant tandem/dispersed repeats. Phylogenetic analyses based on 41 conserved mitochondrial PCGs illustrated its close evolutionary affinity to *C. camphora* and other Laurales species. Collinearity analysis indicated that the mitogenome of *C. longepaniculatum* had undergone extensive genomic rearrangements with its close related species. In conclusion, this research offered comprehensive insights into the organelle genetic characteristics and phylogenetic relationships of *C. longepaniculatum*, serving as an essential reference for subsequent investigations into the genomes of Laurales plants.

## Supplementary Information

Supplementary Material 1.

Supplementary Material 2.

## Data Availability

The raw sequencing data for the Illumina Novaseq 6000 platform and Oxford Nanopore PromethION sequencing platform and the mitogenome sequences have been deposited in NCBI with accession numbers PQ591767.

## Abbreviations

rRNA: Ribosomal RNA
tRNA: Transfer RNA
PCGs: Protein‑coding genes
SSRs: Simple sequence repeats
RSCU: Relative synonymous codon usage ratios
ORFs: Open Reading Frames

## References

[1] Yang Z, Liu B, Yang Y, Ferguson DK. Phylogeny and taxonomy of *Cinnamomum* (Lauraceae). Ecol Evol. 2022;12(10):e9378. 36203627 10.1002/ece3.9378 PMC9526118

[2] Yang Z, Tan C, Wei YM, Rohwer JG, Liu B, Yang Y. Floral morphology and phenology of *Sassafras tzumu* (Lauraceae). BMC Plant Biol. 2022;22(1):327. 35799123 10.1186/s12870-022-03714-6 PMC9264512

[3] Wu ZQ, Liao XZ, Zhang XN, Tembrock LR, Broz A. Genomic architectural variation of plant mitochondria-A review of multichromosomal structuring. J Syst Evol. 2022;60(1):160–8.

[4] Lai C, Wang J, Kan S, Zhang S, Li P, Reeve WG, Wu Z, Zhang Y. Comparative analysis of mitochondrial genomes of Broussonetia spp. (Moraceae) reveals heterogeneity in structure, synteny, intercellular gene transfer, and RNA editing. Front Plant Sci. 2022;13:1052151. 36531410 10.3389/fpls.2022.1052151 PMC9751378

[5] Møller IM, Rasmusson AG, Van Aken O. Plant mitochondria-past, present and future. Plant J. 2021;108(4):912–59. 34528296 10.1111/tpj.15495

[6] Roger AJ, Muñoz-Gómez SA, Kamikawa R. The origin and diversification of mitochondria. Curr Biol. 2017;27(21):R1177–92. 29112874 10.1016/j.cub.2017.09.015

[7] Sagan L. On the origin of mitosing cells. J Theor Biol. 1967;14(3):225–74. 10.1016/0022-5193(67)90079-3 11541392

[8] Birky CW Jr. The inheritance of genes in mitochondria and chloroplasts: laws, mechanisms, and models. Annu Rev Genet. 2001;35:125–48. 11700280 10.1146/annurev.genet.35.102401.090231

[9] Wallace DC, Singh G, Lott MT, Hodge JA, Schurr TG, Lezza AM, Elsas LJ 2nd, Nikoskelainen EK. Mitochondrial DNA mutation associated with Leber’s hereditary optic neuropathy. Science. 1988;242(4884):1427–30. 3201231 10.1126/science.3201231

[10] Petersen G, Cuenca A, Møller IM, Seberg O. Massive gene loss in mistletoe (*Viscum*, Viscaceae) mitochondria. Sci Rep. 2015;5:17588. 26625950 10.1038/srep17588 PMC4667250

[11] Xiang QP, Tang JY, Yu JG, Smith DR, Zhu YM, Wang YR, Kang JS, Yang J, Zhang XC. The evolution of extremely diverged plastomes in Selaginellaceae (lycophyte) is driven by repeat patterns and the underlying DNA maintenance machinery. Plant J. 2022;111(3):768–84. 35648423 10.1111/tpj.15851

[12] Sloan DB, Alverson AJ, Chuckalovcak JP, Wu M, McCauley DE, Palmer JD, Taylor DR. Rapid evolution of enormous, multichromosomal genomes in flowering plant mitochondria with exceptionally high mutation rates. PLoS Biol. 2012;10(1):e1001241. 22272183 10.1371/journal.pbio.1001241 PMC3260318

[13] Sloan DB. One ring to rule them all? Genome sequencing provides new insights into the “master circle” model of plant mitochondrial DNA structure. New Phytol. 2013;200(4):978–85. 24712049 10.1111/nph.12395

[14] Adams KL, Palmer JD. Evolution of mitochondrial gene content: gene loss and transfer to the nucleus. Mol Phylogenet Evol. 2003;29(3):380–95. 14615181 10.1016/s1055-7903(03)00194-5

[15] Cole LW, Guo W, Mower JP, Palmer JD. High and variable rates of repeat-mediated mitochondrial genome rearrangement in a genus of plants. Mol Biol Evol. 2018;35(11):2773–85. 30202905 10.1093/molbev/msy176

[16] Kozik A, Rowan BA, Lavelle D, Berke L, Schranz ME, Michelmore RW, Christensen AC. The alternative reality of plant mitochondrial DNA: One ring does not rule them all. PLoS Genet. 2019;15(8):e1008373. 31469821 10.1371/journal.pgen.1008373 PMC6742443

[17] Sloan DB, Wu Z, Sharbrough J. Correction of persistent errors in Arabidopsis reference mitochondrial genomes. Plant Cell. 2018;30(3):525–7. 29519893 10.1105/tpc.18.00024 PMC5894837

[18] Chen L, Dong X, Huang H, Xu H, Rono PC, Cai X, Hu G. Assembly and comparative analysis of the initial complete mitochondrial genome of *Primulina hunanensis* (Gesneriaceae): a cave-dwelling endangered plant. BMC Genomics. 2024;25(1):322. 38561677 10.1186/s12864-024-10247-9 PMC10983754

[19] Liu GH, Zuo YW, Shan Y, Yu J, Li JX, Chen Y, Gong XY, Liao XM. Structural analysis of the mitochondrial genome of *Santalum album* reveals a complex branched configuration. Genomics. 2024;116(5):110935. 39243912 10.1016/j.ygeno.2024.110935

[20] Shi Y, Chen Z, Jiang J, Wu W, Yu W, Zhang S, Zeng W. The assembly and comparative analysis of the first complete mitogenome of *Lindera aggregata*. Front Plant Sci. 2024;15:1439245. 39290737 10.3389/fpls.2024.1439245 PMC11405213

[21] Che F, Xu M, Yang X, Liu J, Xiao Y, Yang L. An improved approach for the isolation of essential oil from the leaves of *Cinnamomum longepaniculatum* using microwave-assisted hydrodistillation concatenated double-column liquid-liquid extraction. Sep Purif Technol. 2018;195:110–20.

[22] Zhao X, Wei Q, Wu H, Zhou W, Liu M, Yang L, Feng R, Li M. Changes in essential oils content, antioxidant capacity and secondary metabolism in different *Cinnamomum longepaniculatum* varieties. Ind Crop Prod. 2023;192:115996.

[23] Zhao X, Yan Y, Zhou WH, Feng RZ, Shuai YK, Yang L, Liu MJ, He XY, Wei Q. Transcriptome and metabolome reveal the accumulation of secondary metabolites in different varieties of *Cinnamomum longepaniculatum*. BMC Plant Biol. 2022;22(1):243. 35585490 10.1186/s12870-022-03637-2 PMC9116011

[24] Wu D, Zhu P, Wu H, Dai H. Industry development status and prospect of *Cinnamomum longepaniculatum*. OALib. 2022;9:e8616.

[25] Briscoe AG, Hopkins KP, Waeschenbach A. High-throughput sequencing of complete mitochondrial genomes. Methods Mol Biol. 2016;1452:45–64. 27460369 10.1007/978-1-4939-3774-5_3

[26] Feng S, Pozzi A, Stejskal V, Opit G, Yang Q, Shao R, Dowling DK, Li Z. Fragmentation in mitochondrial genomes in relation to elevated sequence divergence and extreme rearrangements. BMC Biol. 2022;20(1):7. 34996453 10.1186/s12915-021-01218-7 PMC8742463

[27] Han F, Bi C, Zhao Y, Gao M, Wang Y, Chen Y. Unraveling the complex evolutionary features of the *Cinnamomum camphora* mitochondrial genome. Plant Cell Rep. 2024;43(7):183. 38922445 10.1007/s00299-024-03256-1

[28] Yang S, Huang J, Qu Y, Zhang D, Tan Y, Wen S, Song Y. Phylogenetic incongruence in an Asiatic species complex of the genus *Caryodaphnopsis* (Lauraceae). BMC Plant Biol. 2024;24(1):616. 38937691 10.1186/s12870-024-05050-3 PMC11212351

[29] Bi C, Sun N, Han F, Xu K, Yang Y, Ferguson DK. The first mitogenome of Lauraceae (*Cinnamomum chekiangense*). Plant Divers. 2023;46(1):144–8. 38343589 10.1016/j.pld.2023.11.001 PMC10851304

[30] Chen S, Zhou Y, Chen Y, Gu J. fastp: an ultra-fast all-in-one FASTQ preprocessor. Bioinformatics. 2018;34(17):i884–90. 30423086 10.1093/bioinformatics/bty560 PMC6129281

[31] Li H. Minimap2: pairwise alignment for nucleotide sequences. Bioinformatics. 2018;34(18):3094–100. 29750242 10.1093/bioinformatics/bty191 PMC6137996

[32] Lu G, Zhang K, Que Y, Li Y. Assembly and analysis of the first complete mitochondrial genome of *Punica granatum* and the gene transfer from chloroplast genome. Front Plant Sci. 2023;14:1132551. 37416882 10.3389/fpls.2023.1132551 PMC10320729

[33] Liu Q, Wu Z, Tian C, Yang Y, Liu L, Feng Y, Li Z. Complete mitochondrial genome of the endangered *Prunus pedunculata* (Prunoideae, Rosaceae) in China: characterization and phylogenetic analysis. Front Plant Sci. 2023;8(14):1266797. 10.3389/fpls.2023.1266797 PMC10753190 38155854

[34] Koren S, Walenz BP, Berlin K, Miller JR, Bergman NH, Phillippy AM. Canu: scalable and accurate long-read assembly via adaptive k-mer weighting and repeat separation. Genome Res. 2017;27(5):722–36. 28298431 10.1101/gr.215087.116 PMC5411767

[35] Langmead B, Salzberg SL. Fast gapped-read alignment with Bowtie 2. Nat Methods. 2012;9(4):357–9. 22388286 10.1038/nmeth.1923 PMC3322381

[36] Wick RR, Judd LM, Gorrie CL, Holt KE. Unicycler: Resolving bacterial genome assemblies from short and long sequencing reads. PLoS Comput Biol. 2017;13(6):e1005595. 28594827 10.1371/journal.pcbi.1005595 PMC5481147

[37] Wick RR, Schultz MB, Zobel J, Holt KE. Bandage: interactive visualization of de novo genome assemblies. Bioinformatics. 2015;31(20):3350–2. 26099265 10.1093/bioinformatics/btv383 PMC4595904

[38] Tillich M, Lehwark P, Pellizzer T, Ulbricht-Jones ES, Fischer A, Bock R, Greiner S. GeSeq-versatile and accurate annotation of organelle genomes. Nucleic Acids Res. 2017;45(W1):W6-11. 28486635 10.1093/nar/gkx391 PMC5570176

[39] Chan PP, Lowe TM. tRNAscan-SE: Searching for tRNA Genes in Genomic Sequences. Methods Mol Biol. 2019;1962:1–14. 31020551 10.1007/978-1-4939-9173-0_1 PMC6768409

[40] Greiner S, Lehwark P, Bock R. OrganellarGenomeDRAW (OGDRAW) version 1.3.1: expanded toolkit for the graphical visualization of organellar genomes. Nucleic Acids Res. 2019;47(W1):W59–64. 30949694 10.1093/nar/gkz238 PMC6602502

[41] Beier S, Thiel T, Münch T, Scholz U, Mascher M. MISA-web: a web server for microsatellite prediction. Bioinformatics. 2017;33(16):2583–5. 28398459 10.1093/bioinformatics/btx198 PMC5870701

[42] Benson G. Tandem repeats finder: a program to analyze DNA sequences. Nucleic Acids Res. 1999;27(2):573–80. 9862982 10.1093/nar/27.2.573 PMC148217

[43] Krzywinski M, Schein J, Birol I, Connors J, Gascoyne R, Horsman D, Jones SJ, Marra MA. Circos: an information aesthetic for comparative genomics. Genome Res. 2009;19(9):1639–45. 19541911 10.1101/gr.092759.109 PMC2752132

[44] Altschul SF, Gish W, Miller W, Myers EW, Lipman DJ. Basic local alignment search tool. J Mol Biol. 1990;215(3):403–10. 2231712 10.1016/S0022-2836(05)80360-2

[45] Katoh K, Standley DM. MAFFT multiple sequence alignment software version 7: improvements in performance and usability. Mol Biol Evol. 2013;30:772–80. 23329690 10.1093/molbev/mst010 PMC3603318

[46] Santorum JM, Darriba D, Taboada GL, Posada D. jmodeltest.org: selection of nucleotide substitution models on the cloud. Bioinformatics. 2014;30(9):1310–1. 24451621 10.1093/bioinformatics/btu032 PMC3998143

[47] Togkousidis A, Kozlov OM, Haag J, Höhler D, Stamatakis A. Adaptive RAxML-NG: Accelerating Phylogenetic Inference under Maximum Likelihood using Dataset Difficulty. Mol Biol Evol. 2023;40(10):msad227. 37804116 10.1093/molbev/msad227 PMC10584362

[48] Li W, Godzik A. Cd-hit: a fast program for clustering and comparing large sets of protein or nucleotide sequences. Bioinformatics. 2006;22(13):1658–9. 16731699 10.1093/bioinformatics/btl158

[49] Marçais G, Delcher AL, Phillippy AM, Coston R, Salzberg SL, Zimin A. MUMmer4: A fast and versatile genome alignment system. PLoS Comput Biol. 2018;14(1):e1005944. 29373581 10.1371/journal.pcbi.1005944 PMC5802927

[50] Jiang M, Ni Y, Zhang J, Li J, Liu C. Complete mitochondrial genome of Mentha spicata L. reveals multiple chromosomal configurations and RNA editing events. Int J Biol Macromol. 2023;251:126257. 37573900 10.1016/j.ijbiomac.2023.126257

[51] Kurtz S, Choudhuri JV, Ohlebusch E, Schleiermacher C, Stoye J, Giegerich R. REPuter: the manifold applications of repeat analysis on a genomic scale. Nucleic Acids Res. 2001;29(22):4633–42. 11713313 10.1093/nar/29.22.4633 PMC92531

[52] Mower JP. Variation in protein gene and intron content among land plant mitogenomes. Mitochondrion. 2020;53:203–13. 32535166 10.1016/j.mito.2020.06.002

[53] Zardoya R. Recent advances in understanding mitochondrial genome diversity. F1000Res. 2020;17(9):F1000. 10.12688/f1000research.21490.1 PMC7194472 32399193

[54] Oldenburg DJ, Bendich AJ. DNA maintenance in plastids and mitochondria of plants. Front Plant Sci. 2015;6:883. 26579143 10.3389/fpls.2015.00883 PMC4624840

[55] Wynn EL, Christensen AC. Repeats of unusual size in plant mitochondrial genomes: identification, incidence and evolution. G3 (Bethesda). 2019;9(2):549–59. 30563833 10.1534/g3.118.200948 PMC6385970

[56] Backert S, Nielsen BL, Borner T. The mystery of the rings: structure and replication of mitochondrial genomes from higher plants. Trends Plant Sci. 1997;2(97):477–83.

[57] Yu R, Chen X, Long L, Jost M, Zhao R, Liu L, Mower JP, dePamphilis CW, Wanke S, Jiao Y. *De novo* assembly and comparative analyses of mitochondrial genomes in Piperales. Genome Biol Evol. 2023;15(3):evad041. 36896589 10.1093/gbe/evad041 PMC10036691

[58] Hershberg R, Petrov DA. Selection on codon bias. Annu Rev Genet. 2008;42:287–99. 18983258 10.1146/annurev.genet.42.110807.091442

[59] Passamonti M, Calderone M, Delpero M, Plazz F. Clues of *in vivo* nuclear gene regulation by mitochondrial short non-coding RNAs. Sci Rep. 2020;10(1):8219. 32427953 10.1038/s41598-020-65084-z PMC7237437

[60] Parvathy ST, Udayasuriyan V, Bhadana V. Codon usage bias. Mol Biol Rep. 2022;49(1):539–65. 34822069 10.1007/s11033-021-06749-4 PMC8613526

[61] Schuster J, Knill T, Reichelt M, Gershenzon J, Binder S. Branched-chain aminotransferase4 is part of the chain elongation pathway in the biosynthesis of methionine-derived glucosinolates in *Arabidopsis*. Plant Cell. 2006;18(10):2664–79. 17056707 10.1105/tpc.105.039339 PMC1626624

[62] Sheng W, Deng J, Wang C, Kuang Q. The garden asparagus (Asparagus officinalis L.) mitochondrial genome revealed rich sequence variation throughout whole sequencing data. Front Plant Sci. 2023;14:1140043. 37051082 10.3389/fpls.2023.1140043 PMC10084930

[63] Jiang M, Ni Y, Li J, Liu C. Characterisation of the complete mitochondrial genome of *Taraxacum mongolicum* revealed five repeat-mediated recombinations. Plant Cell Rep. 2023;42(4):775–89. 36774424 10.1007/s00299-023-02994-y

[64] Ma Q, Wang Y, Li S, Wen J, Zhu L, Yan K, Du Y, Ren J, Li S, Chen Z, Bi C, Li Q. Assembly and comparative analysis of the first complete mitochondrial genome of *Acer truncatum* Bunge: a woody oil-tree species producing nervonic acid. BMC Plant Biol. 2022;22(1):29. 35026989 10.1186/s12870-021-03416-5 PMC8756732

[65] Gualberto JM, Mileshina D, Wallet C, Niazi AK, Weber-Lotfi F, Dietrich A. The plant mitochondrial genome: dynamics and maintenance. Biochimie. 2014;100:107–20. 24075874 10.1016/j.biochi.2013.09.016

[66] Bi C, Lu N, Xu Y, He C, Lu Z. Characterization and analysis of the mitochondrial genome of common bean (*Phaseolus vulgaris*) by comparative genomic approaches. Int J Mol Sci. 2020;21(11):3778. 32471098 10.3390/ijms21113778 PMC7312688

[67] Yang H, Li W, Yu X, Zhang X, Zhang Z, Liu Y, Wang W, Tian X. Insights into molecular structure, genome evolution and phylogenetic implication through mitochondrial genome sequence of *Gleditsia sinensis*. Sci Rep. 2021;11(1):14850. 34290263 10.1038/s41598-021-93480-6 PMC8295344

[68] Wang X, Zhang R, Yun Q, Xu Y, Zhao G, Liu J, Shi S, Chen Z, Jia L. Comprehensive analysis of complete mitochondrial genome of Sapindus mukorossi Gaertn.: an important industrial oil tree species in China. Ind Crop Prod. 2021;174:114210.

[69] Cheng Y, He X, Priyadarshani SVGN, Wang Y, Ye L, Shi C, Ye K, Zhou Q, Luo Z, Deng F, Cao L, Zheng P, Aslam M, Qin Y. Assembly and comparative analysis of the complete mitochondrial genome of *Suaeda glauca*. BMC Genomics. 2021;22(1):167. 33750312 10.1186/s12864-021-07490-9 PMC7941912

[70] Wang XC, Chen H, Yang D, Liu C. Diversity of mitochondrial plastid DNAs (MTPTs) in seed plants. Mitochondrial DNA A DNA Mapp Seq Anal. 2018;29(4):635–42. 28573928 10.1080/24701394.2017.1334772

[71] Sibbald SJ, Lawton M, Archibald JM. Mitochondrial genome evolution in *Pelagophyte Algae*. Genome Biol Evol. 2021;13(3):evab018. 33675661 10.1093/gbe/evab018 PMC7936722

[72] Richardson AO, Rice DW, Young GJ, Alverson AJ, Palmer JD. The “fossilized” mitochondrial genome of *Liriodendron tulipifera*: ancestral gene content and order, ancestral editing sites, and extraordinarily low mutation rate. BMC Biol. 2013;11:29. 23587068 10.1186/1741-7007-11-29 PMC3646698

[73] Anderson BM, Krause K, Petersen G. Mitochondrial genomes of two parasitic *Cuscuta* species lack clear evidence of horizontal gene transfer and retain unusually fragmented *ccmF*_*C*_ genes. BMC Genomics. 2021;22(1):816. 34772334 10.1186/s12864-021-08105-z PMC8588681

[74] Choi KS, Park S. Complete Plastid and mitochondrial genomes of *Aeginetia indica* reveal intracellular gene transfer (IGT), horizontal gene transfer (HGT), and cytoplasmic male sterility (CMS). Int J Mol Sci. 2021;22(11):6143. 34200260 10.3390/ijms22116143 PMC8201098

[75] Bi C, Paterson AH, Wang X, Xu Y, Wu D, Qu Y, Jiang A, Ye Q, Ye N. Analysis of the complete mitochondrial genome sequence of the diploid cotton *Gossypium raimondii* by comparative genomics approaches. Biomed Res Int. 2016;2016:5040598. 27847816 10.1155/2016/5040598 PMC5099484

[76] Lukeš J, Kaur B, Speijer D. RNA editing in mitochondria and plastids: weird and widespread. Trends Genet. 2021;37(2):99–102. 33203574 10.1016/j.tig.2020.10.004

[77] Ou T, Wu Z, Tian C, Yang Y, Li Z. Complete mitochondrial genome of *Agropyron cristatum* reveals gene transfer and RNA editing events. BMC Plant Biol. 2024;24(1):830. 39232676 10.1186/s12870-024-05558-8 PMC11373303

[78] Tsai HC, Hsieh CH, Hsu CW, Hsu YH, Chien LF. Cloning and organelle expression of Bamboo mitochondrial Complex I subunits Nad1, Nad2, Nad4, and Nad5 in the Yeast *Saccharomyces cerevisiae*. Int J Mol Sci. 2022;23(7):4054. 35409414 10.3390/ijms23074054 PMC8999482

[79] Small ID, Schallenberg-Rüdinger M, Takenaka M, Mireau H, Ostersetzer-Biran O. Plant organellar RNA editing: what 30 years of research has revealed. Plant J. 2020;101(5):1040–56. 31630458 10.1111/tpj.14578

[80] Sloan DB, MacQueen AH, Alverson AJ, Palmer JD, Taylor DR. Extensive loss of RNA editing sites in rapidly evolving Silene mitochondrial genomes: selection vs. retroprocessing as the driving force. Genetics. 2010;185(4):1369–80. 20479143 10.1534/genetics.110.118000 PMC2927763

[81] Handa H. The complete nucleotide sequence and RNA editing content of the mitochondrial genome of rapeseed (Brassica napus L.): comparative analysis of the mitochondrial genomes of rapeseed and Arabidopsis thaliana. Nucleic Acids Res. 2003;31(20):5907–16. 14530439 10.1093/nar/gkg795 PMC219474

[82] Galtier N. The intriguing evolutionary dynamics of plant mitochondrial DNA. BMC Biol. 2011;9:61. 21951676 10.1186/1741-7007-9-61 PMC3181201

[83] Group TAP, Chase MW, Christenhusz MJM, Fay MF, Byng JW, Judd WS, Soltis DE, Mabberley DJ, Sennikov AN, Soltis PS, Stevens PF. An update of the Angiosperm Phylogeny Group classification for the orders and families of flowering plants: APG IV. Bot J Linn Soc. 2016;181:1–20.

[84] Liu LX, Du YX, Folk RA, Wang SY, Soltis DE, Shang FD, Li P. Plastome evolution in Saxifragaceae and multiple plastid capture events involving *Heuchera* and *Tiarella*. Front Plant Sci. 2020;11:361. 32391025 10.3389/fpls.2020.00361 PMC7193090

[85] Yin H, Akimoto M, Kaewcheenchai R, Sotowa M, Ishii T, Ishikawa R. Inconsistent diversities between nuclear and plastid genomes of AA genome species in the genus Oryza. Genes Genet Syst. 2015;90(5):269–81. 26687860 10.1266/ggs.14-00063

[86] Zhu W, Zhang D, Xu W, Gan Y, Huang J, Liu Y, Tan Y, Song Y, Xin P. Comparative genomics and phylogenetic analysis of mitochondrial genomes of Neocinnamomum. BMC Plant Biol. 2025;25(1):289. 40045193 10.1186/s12870-025-06238-x PMC11883965

