# Supplementary material for: Assembly and comparative analysis of the complete mitochondrial genome of the spice plant Cinnamomum longepaniculatum
Source: BMC Plant Biol. 2025 Jul 16;25:916. doi: 10.1186/s12870-025-06839-6 (PMC12265261; doi:10.1186/s12870-025-06839-6)
Supplement: Supplementary file 2 — Supplementary Material 2. [file 12870_2025_6839_MOESM2_ESM.docx]

**M**


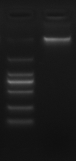


Fig.1 Agarose gel electrophoresis of the total DNA sample prepared for sequencing on the Illumina NovaSeq 6000 platform

Note: The lane on the left was the DL2000 DNA Marker, ranging from 100bp to 2000bp, while the lane on the right presented the total DNA of *Cinnamomum longepaniculatum*. The gel concentration was set to 1%, the applied voltage was 120V, and the electrophoresis duration was 25 minutes.

The total DNA was extracted using a universal plant DNA extraction kit. Initial DNA quality assessment was performed via agarose gel electrophoresis and one drop. The results indicated that the sample concentration was 8.55 ng/μl, the extracted sample volume was 40 μl, and the OD260/280 ratio was 1.70. The quality of the samples satisfied the requirements for sequencing.


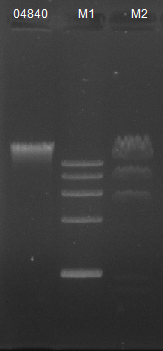


Fig.2 Agarose gel electrophoresis of the mitogenome sample prepared for sequencing on the Nanopore PromethION platform

Note: M1 denoted the 15kb DNA Marker, with specific bands at 15000 bp, 10000 bp, 7500 bp, 5000 bp, 2500 bp, 1000 bp and 250 bp. M2 represents the λDNA /HindIII, yielding fragments of 23130 bp, 9416 bp, 6557 bp, 4361 bp, 2322 bp, 2027 bp, 564 bp. The gel concentration was set to 0.7%, the applied voltage was 100V, and the electrophoresis duration was 60 minutes.

DNA was extracted from *Cinnamomum longepaniculatum* samples using the CTAB method and subsequently purified with the 1330230 kit. Nanodrop analysis revealed a DNA concentration of 43.8 ng/μl with an OD260/280 ratio of 1.87. Qubit analysis indicated a DNA concentration of 42.4 ng/μl, resulting in an Nc/Qc (Nanodrop/Qubit) ratio of 1.03. Comprehensive quality assessments demonstrated that the DNA samples achieved Grade A quality, fulfilling the technical requirements for sequencing on the Nanopore PromethION platform.
